# Effects of AKAP5 Pro100Leu Genotype on Working Memory for Emotional Stimuli

**DOI:** 10.1371/journal.pone.0055613

**Published:** 2013-01-29

**Authors:** Sylvia Richter, Xenia Gorny, Judith Machts, Gusalija Behnisch, Torsten Wüstenberg, Maike C. Herbort, Thomas F. Münte, Constanze I. Seidenbecher, Björn H. Schott

**Affiliations:** 1 Department of Clinical Psychology, University of Salzburg, Salzburg, Austria; 2 Leibniz Institute for Neurobiology, Magdeburg, Germany; 3 Department of Neurology, University of Magdeburg, Magdeburg, Germany; 4 Department of Psychiatry, Charité – Universitätsmedizin Berlin, Charité Campus Mitte, Berlin, Germany; 5 Department of Neurology, University of Lübeck, Lübeck, Germany; University of Turin, Italy

## Abstract

Recent investigations addressing the role of the synaptic multiadaptor molecule AKAP5 in human emotion and behavior suggest that the AKAP5 Pro100Leu polymorphism (rs2230491) contributes to individual differences in affective control. Carriers of the less common Leu allele show a higher control of anger as indicated by behavioral measures and dACC brain response on emotional distracters when compared to Pro homozygotes. In the current fMRI study we used an emotional working memory task according to the n-back scheme with neutral and negative emotional faces as target stimuli. Pro homozygotes showed a performance advantage at the behavioral level and exhibited enhanced activation of the amygdala and fusiform face area during working memory for emotional faces. On the other hand, Leu carriers exhibited increased activation of the dACC during performance of the 2-back condition. Our results suggest that AKAP5 Pro100Leu effects on emotion processing might be task-dependent with Pro homozygotes showing lower control of emotional interference, but more efficient processing of task-relevant emotional stimuli.

## Introduction

Over the past decade, a growing number of studies have made use of genetics to characterize molecular influences on cognition and behavior, thereby also contributing new insights on individual differences. Common genetic variations in the population contribute to the inter-individual variability in cognitive functions like attention or memory as well as affective domains like trait-anxiety, motivation, impulsivity and emotion regulation [1]–[7]. The use of functional neuroimaging has elucidated the neural mechanisms that mediate the influence of specific genes on cognitive processing by linking genetic variations to individual differences in activation patterns of the prefrontal cortex (PFC) and associated neural circuits [1], [8]. On the other hand, genetic variations involved in emotion-related phenomena have been shown to affect the response of brain structures relevant for emotion processing like the amygdala and the rostral cingulate cortex [9], [10].

Previous behavioral and neuroimaging studies of emotional processing have emphasized the role of serotonergic signaling [4], [9]–[11], while investigations of genetic influences on executive functions have focused on molecular determinants of prefrontal dopamine, particularly catechol-O-methyltransferase (COMT) with its well-studied single nucleotide polymorphism Val108/158Met [1], [6].

The term emotion regulation has been used to describe a component of the complex interplay of cognitive and emotional processes, namely the control of emotions by different cognitive strategies [12]. These modulation processes are mediated by PFC-amygdala interactions [13]–[16]. Erk and colleagues could demonstrate a valence-specific regulation effect in participants viewing emotionally salient pictures. In that study, high cognitive effort during emotion regulation was associated with reduced activity in the amygdala and the ventral striatum, which was mediated by different prefrontal regions [14].

Several studies have investigated genetic modifiers of prefrontal-limbic interactions, although few have focused on emotion regulation directly. Some genetic variations associated with individual differences in emotion processing have also been linked to alterations in prefrontal fMRI responses to emotional stimuli, like the COMT Val108/158Met polymorphism [17] or to structural and functional alterations in frontolimbic circuits associated with cognitive control of emotions, like the serotonin (5-HT) transporter gene linked polymorphic region (5-HTTLPR) variants [18], [19]. Notably, the increased amygdala activation during emotion processing that is commonly observed in carriers of the 5-HTTLPR short allele [9], [20], can be suppressed by cognitive processes related to emotion regulation [11]. Additionally, a common monoamine oxidase A (MAOA) gene polymorphism is known to influence brain circuits involved in emotion processing and emotion regulation [20], [21], although most studies have not addressed its effects on emotion regulation specifically. Despite these studies implicating monoamine signaling in frontolimbic circuits [20], the genetics of emotion regulation is still not well understood.

One reason for the apparent difficulty to assess genetic influences on inter-individual variability in the interaction between cognitive and emotional processing might be the differential influences of distinct neurotransmitter systems on subprocesses of cognitive and affective functions. Dopamine primarily affects motivational processing in the striatum [22]–[24] and PFC-dependent executive functions [25], [26] but also emotional reactivity [17], [27]. Pronounced serotonergic influences have been reported for amygdala-dependent emotion processing [4] and hippocampus-dependent long-term memory [28], [29]. Pharmacological evidence has further implicated noradrenergic neurotransmission in emotion processing [30] and attention [31]. Importantly, in addition to polymorphisms in the monoamine systems, genetic variations affecting the fine-tuning of glutamatergic neurotransmission can also influence PFC-dependent cognition [32], particularly in interaction with variations in the dopaminergic pathway [33].

Given these partly domain-specific influences of genetic polymorphisms in monoaminergic and glutamatergic pathways on distinct aspects of cognition, emotion, and behavior, we hypothesized that inter-individual variability of the cognitive control of emotional processes might in part be mediated by genetic variations in adaptor molecules that mediate the integration of monoaminergic and glutamatergic signalling. The synaptic multiadaptor molecule A-kinase anchoring protein 5 (AKAP5, AKAP79/150) forms part of the intracellular signalling cascades of beta-adrenergic, dopaminergic and serotonergic signalling by linking their G-protein-coupled receptors (GPCRs) to intracellular effector molecules and to postsynaptic components of glutamatergic neurotransmission [34]–[36]. Thus, it is a promising candidate molecule for genetic variability of emotion-cognition interactions. Animal studies have demonstrated a role of AKAP5-dependent signalling in complex behavior, including fear memory [37], [38]. We could recently demonstrate that, in healthy young human participants, a functional genetic polymorphism of the AKAP5 gene, Pro100Leu (NCBI accession #: rs2230491), affects anger control and physical aggressive behavior. Carriers of the less common Leu allele show lower physical aggression and higher anger control [39]. On the neural level, Leu carriers showed a more effective dACC-mediated control of emotional interference in a modified flanker task with emotional distracters (faces) when compared to Pro homozygotes. On the other hand, Pro homozygotes exhibited increased orbitofrontal cortex (OFC) activation during emotional interference, which might reflect a more sensitive reaction to the emotional stimulus components and a stronger activation of the ventral affective processing system.

In the present study, we sought to assess how neural mechanisms of implicit emotion regulation are modulated by AKAP5 Pro100Leu when emotional stimuli are targets rather than task-irrelevant distracters. To this end, we performed a functional magnetic resonance imaging (fMRI) study using a working memory task (n-back) with neutral and emotional faces as target stimuli (EFNBACK) [40]. In the original EFNBACK task, target faces were flanked by emotional or neutral distracters. These were omitted in a simplified EFNBACK task used in the present study in order to avoid interfering effects previously observed in a flanker task with emotional distracters [39]. We hypothesized that, because of their presumably higher emotional reactivity, Pro homozygous subjects might show an advantage in the encoding and maintenance of emotional faces and hence perform the working memory task more successfully. At a neural level, their better performance might be reflected by an increased fMRI response of the amygdala and possibly additional components of the ventral affective processing system.

## Methods

### Participants

34 young, neurologically and psychiatrically healthy volunteers (18 Pro homozygotes, including 8 women; 16 heterozygous Leu carriers, including 9 women) took part in the fMRI study after exclusion of contraindications. All participants gave written informed consent in accordance with the Declaration of Helsinki and received financial compensation for participation. The work was approved by the Ethics Committee of the University of Magdeburg, Faculty of Medicine.

### Genotyping

Genotyping was performed on DNA extracted from blood leukocytes using PCR-based allele-specific restriction analysis [39]. Briefly, the DNA fragment containing the AKAP5 Pro100Leu polymorphism (Chr 14q21-24) was amplified using the primers AKAP5_100-f (5'-GCT TCT GAT CAG CCA GAG CCC AC-3') and AKAP5_100-r (5'-GCT TCT TCC TGG ACT TTG ATG CTG CAG-3') and standard Taq polymerase (Qiagen and Fermentas). PCR products were digested with the restriction endonuclease AluI (Fermentas), yielding two fragments (174+98 bp) for the Leu allele or a single fragment (272 bp) for the Pro allele. DNA fragments were separated on ethidium bromide-stained agarose gels and visualized under UV light.

### Functional MRI experiment

#### Experimental procedure

The goal of this fMRI study was to assess how AKAP5 Pro100Leu affects emotional salience of target stimuli in working memory. The N-back working memory task has been shown to robustly recruit lateral premotor cortex, dorsal cingulate and medial premotor cortex, dorsolateral and ventrolateral prefrontal cortex, frontal poles, and medial and lateral posterior parietal cortex [41]. Moreover, negative emotional salience has been demonstrated to affect working memory performance [42].

**Figure 1:**
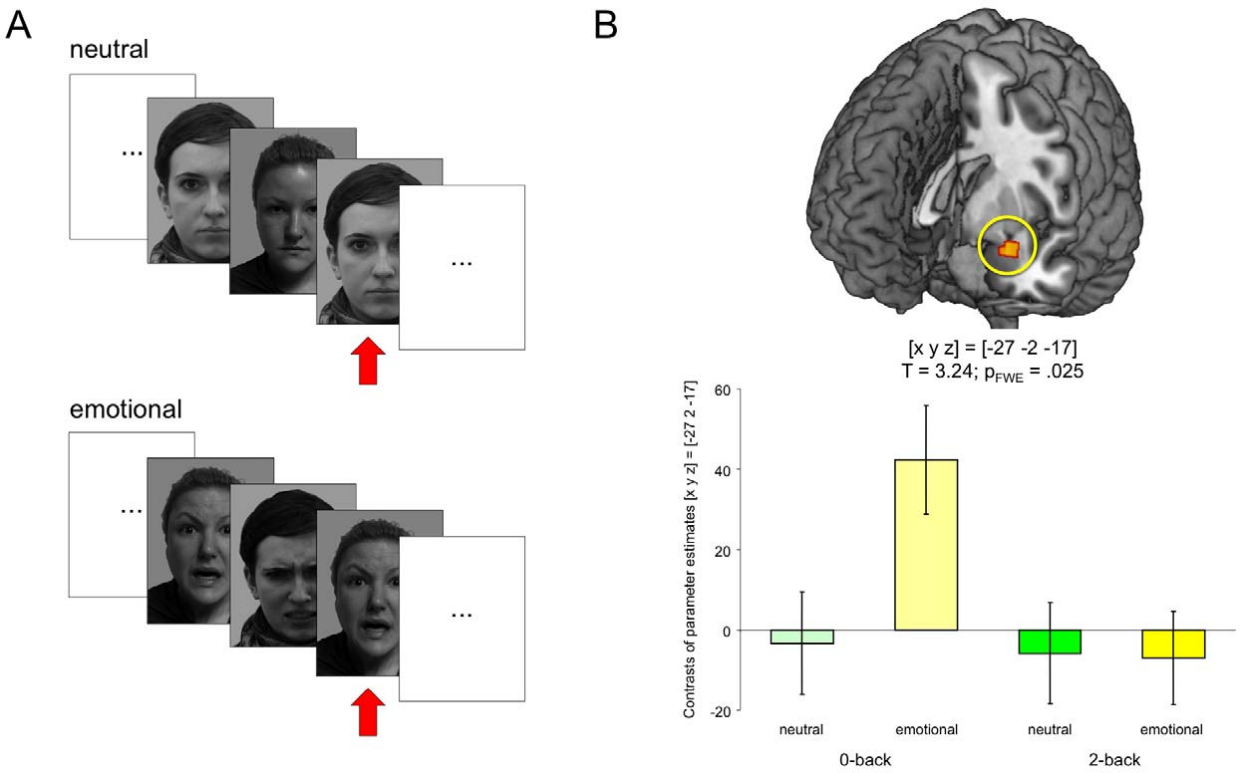
Experimental design und corresponding functional MRI correlates of working memory for emotional stimuli. (**A**) Schematic illustration of the stimulation. Target stimuli in the 2-back condition are highlighted by red arrows. See Methods section for details. *Note:* The face stimuli depicted in the figure were not presented in the original study and are displayed for illustrative purposes. The persons depicted gave written informed consent to have their photograph displayed here as outlined in the PLoS consent form. (**B**) Emotional stimuli elicited an amygdala response during 0-back performance, whereas no reliable amygdala activation was observed during the 2-back task across the whole cohort. Bar plots depict contrasts of parameter estimates and standard errors.

The trial structure of our simplified EFNBACK task is depicted in figure 1A. All trials started with the presentation of a cue indicating the upcoming condition (2-back or 0-back). During both conditions, faces were presented sequentially (presentation time  =  1 s, followed by a fixation cross for 500 ms). In the baseline condition (0-back), participants were instructed to respond via button press whenever a previously defined target face was presented. In the task condition (2-back), participants were instructed to respond whenever the face was the same as the face presented 2 trials ago. In total, eight blocks of each condition were presented, each block lasting 28 s. In four blocks (two 2-back and two 0-back), faces had neutral expressions, and in the other four blocks, faces had negative emotional expressions (angry or fearful). Faces were taken from the Karolinska Directed Emotional Faces database (KDEF) [43] and converted into grayscale. Button press responses were recorded and scored separated into target responses (hits) and non-target responses (false alarms).

#### Image acquisition

Functional magnetic resonance imaging was performed using a GE Signa 1.5 T magnetic resonance system (General Electric) and a standard circularly polarized head coil. A single session of 316 echo-planar images (EPIs) was acquired in an interleaved manner [23 axial slices; voxel size  =  3.13×3.13×4 mm + 1 mm gap; TR  =  2 sec; TE  =  35 msec; odd numbers first]. Additionally, a co-planar proton-density (PD)-weighted MR image was acquired and used for coregistration to improve spatial normalization.

#### Data processing and analysis

Statistical analyses were performed using SPM8b (Statistical Parametric Mapping, Wellcome Department of Imaging Neuroscience, London, UK). EPIs were corrected for acquisition delay and head motion. The co-planar PD-weighted image was coregistered to the mean EPI image obtained from motion correction and, segmented using the unified segmentation approach as provided by SPM. Segments were warped to the standard space as defined by the Montreal Neurological Institute (MNI) by affine and nonlinear coregistration to the brain template of the International consortium for Brain Mapping (ICBM-Template implemented in SPM). The normalization parameters obtained from this procedure were used for spatial normalization of EPIs. Finally, normalized EPIs were smoothed with an isotropic Gaussian kernel (8 mm FWHM).

Statistical analysis was performed using a two-stage mixed-effects model. In the first stage, stimulus-dependent neural activity was modeled by box-car functions (duration  =  28 s) at the onset of each block in the four conditions of interest ([2-back & 0-back] * [emotional & neutral]) and convolved with the canonical hemodynamic response function (HRF) provided by SPM. The resulting time courses were downsampled for each scan to form regressors in a general linear model (GLM). The GLM contained separate regressors for the four conditions of interest, covariates of no interest for the six rigid-body-movement parameters determined from motion correction (to capture residual fluctuations in MR signal due to movement*susceptibility interaction), and single constant representing the mean over scans. Parameters for each regressor were estimated by a Restricted Maximum Likelihood (ReML) fit. Based on the resulting parameter estimates linear contrast images were computed for the effects of interest. In the second stage, these contrast images were submitted to separate random effects analyses. Specifically, the contrast images for the factors emotional salience (angry or fearful vs. neutral) and working memory load (2-back/0-back) were incorporated in two-way analyses of variance (ANOVA) with genotype (Pro/Pro vs. Leu carriers) as between-subjects factor and gender as covariate. *Post hoc* two-sample t-tests were computed to assess between-group effects. Given the expression of AKAP5 in the amygdala [44], which plays a key role in emotion processing [9], [45]–[55], and the known described cortical-amygdalar interactions associated with emotion regulation, we focused our analyses the left and right amygdalae. ROI-based analyses were also conducted for other brain structures involved in cognition-emotion interactions, namely the fusiform face area (FFA), the insula, and the rostral and dorsal anterior cingulate cortex (rACC, dACC). All analyses were performed using literature-based probabilistic ROIs that were generated based on previously published fMRI studies using a previously described algorithm [56], [57] (Detailed coordinate lists and ROIs in NIFTI format are available from the authors upon request). The significance level was set to p<05, family-wise error (FWE)-corrected for the ROI volumes. T-contrasts testing for directionality in conditions of specific interest were masked with the appropriate F-contrasts (thresholded at p<05, uncorrected) of the corresponding main effects or interactions.

## Results

### Behavioral results

**Figure 2:**
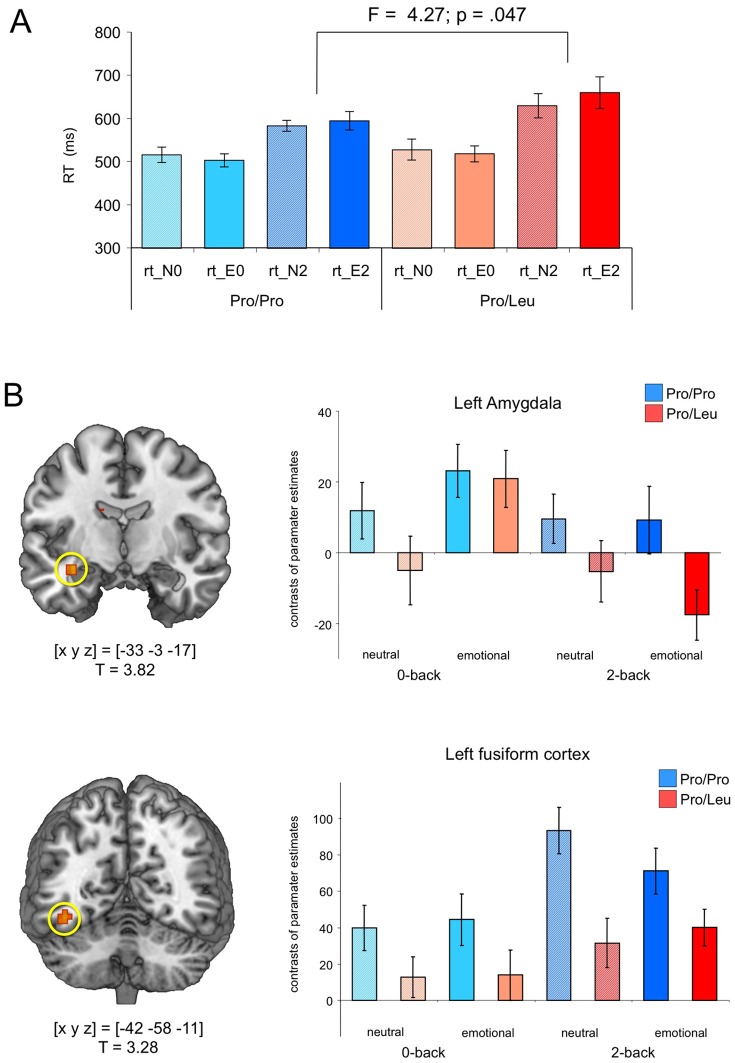
Genotype-dependent modulation of amygdala activation. (**A**) Behavioral results. Leu carriers showed longer reaction times during 2-back performance, particularly in the emotional condition. (**B**) Top: Amygdala activation during 2-back performance in the emotional condition was observed in Pro homozygotes, but was largely absent in Leu carriers. There was also a trend for higher amygdala activation in the neutral and emotional 0-back conditions. Bottom: Pro homozygotes also exhibited stronger FFA activation in all conditions. Bar plots depict contrasts of parameter estimates and standard errors.

Demographic and behavioral data are displayed in Table 1. Neither gender distribution (*X* ^2^ = 0.47; p = 473) nor age (t = 0.28; p = 780) differed significantly between both groups. In line with earlier studies [41], [58], [59], higher working memory load (2-back vs. 0-back) was associated with fewer hits [F_1,32_ = 57.67; p<001; 2-way ANOVA for repeated measures with AKAP5 genotype as between-subjects factor] and higher false alarm rates [F_1,32_ = 71.47; p<001], but neither hits nor false alarms were influenced by emotional salience or AKAP5 genotype [all p>138]. Reaction times (RTs) were significantly longer in the 2-back condition across the whole cohort [F_1,32_ = 98.06; p<001], and Leu carriers showed longer RTs in the 2-back conditions relative to Pro homozygotes (Figure 2A; Table 1) [working memory load by genotype interaction: F_1,32_ = 4.27; p = 047]. Moreover, there was a trend for an interaction between working memory load and emotional salience [F_1,32_ = 3.67; p = 065].

**Table 1:** Demographic and behavioral data of the fMRI experiment.

Variable	Pro/Pro		Leu carriers	
	*M*	*SD*	*M*	*SD*
**A**ge	23.7	2.1	23.9	2.2
**Hits**				
*** 0-back***				
emotional	100	0.0	98.8	4.7
neutral	100	0.0	98.8	3.4
*** 2-back***				
emotional	88.4	12.4	82.3	16.1
neutral	86.0	10.8	82.1	9.4
**False alarms**				
*** 0-back***				
emotional	0.0	0.0	0.7	1.2
neutral	0.2	0.6	0.7	2.2
*** 2-back***				
emotional	5.4	4.3	5.3	3.7
neutral	4.9	5.5	5.9	4.4
**Reaction times**				
*** 0-back***				
emotional	503	65	519	75
neutral	516	75	528	98
*** 2-back***				
emotional	595	93	661	146
neutral	584	53	630	112

Mean error rates in per cent and reaction times +/− standard deviation are shown.

Abbreviations: M: mean; SD: standard deviation; RT: reaction time.

### Functional MRI results

#### Effects of working memory load

Irrespective of stimulus valence, performance of the 2-back relative to the 0-back condition was associated with increased activation of a fronto-parietal network, including ventrolateral and dorsolateral PFC, dACC, and posterior parietal cortex (Table 2). This pattern replicates previous results of studies using the N-back task [25], [33], [41]. Conversely, the 0-back condition was associated with enhanced BOLD responses in Default Mode Network structures such as the posterior cingulate and the medial PFC when compared to the 2-back condition.

**Table 2:** Neural correlates of working memory load.

2-back versus 0-back					
Region	x	y	z	SPM T	p (FWE-corr.)
Right ventrolateral PFC (BA 47)	30	23	−2	5.76	0.001
Left dorsolateral PFC (BA 46)	−45	29	28	5.80	0.001
	−30	−1	52	8.24	0.000
Right dorsolateral PFC (BA 6, 46)	30	2	52	8.64	0.000
	42	38	28	6.13	0.000
Left frontopolar cortex (BA 10)	−33	47	13	7.25	0.001
Left premotor cortex (BA 6)	−39	2	25	5.55	0.001
Left insula (BA 13)	−30	20	1	6.04	0.000
Left dACC (BA 32)	−6	17	43	8.97	0.000
Right dACC (BA 32)	12	32	22	6.10	0.000
Left posterior parietal cortex (BA 7, 40)	−30	−49	37	6.65	0.000
	−9	−67	49	8.55	0.000
	−42	−43	37	6.41	0.000
Right posterior parietal cortex (BA 7, 40)	18	−67	52	8.78	0.000
	42	−40	43	6.92	0.000
Left precuneus (BA 19)	−27	−73	31	5.99	0.001
Thalamus	9	−22	10	6.64	0.001
Left cerebellum	−36	−67	−32	8.63	0.001
Right cerebellum	9	−79	−26	5.37	0.002
	3	−34	−17	6.82	0.001
Brain stem	6	−28	−8	7.60	0.001
Left hypothalamus	−9	−7	−5	4.95	0.010
Left caudate	−15	5	13	6.14	0.001
Left pallidum	−12	2	1	5.76	0.001
Right pallidum	12	−4	1	5.36	0.002

Activations related to working memory load (2-back vs. 0-back), separated by stimulus valence (neutral vs. emotional). All activations were whole-brain FWE-corrected (p<.05). Abbreviations: BA: Brodmann area; dACC: dorsal anterior cingulate cortex; PFC: prefrontal cortex.

#### Interaction of working memory load and emotional processing

A direct comparison of emotional versus neutral face stimuli in the 0-back inclusively masked with the interaction contrast N-back by emotional salience condition yielded an activation of the left amygdala, ([x y z]  =  [−27 −2 −17]; T = 3.24; p = 025, small-volume FWE-corrected for the corresponding literature-based probabilistic ROI; see methods section), whereas no amygdala activity was observed in the whole sample during the 2-back condition (Figure 1B). No other brain regions exhibited a reliable genotype-independent interaction of task difficulty and emotional salience.

#### Effects of AKAP5 Pro100Leu on emotion processing during working memory performance

As outlined in the methods section, the primary aim of this study was to investigate how AKAP5 Pro100Leu influences the amygdala response under conditions of high working memory load. We therefore conducted a ROI analysis in the left and right amygdalae, using probabilistic ROIs based on previous fMRI literature [56], [57]. When compared to Leu carriers, Pro homozygotes showed significantly higher activation of the left amygdala in the emotional 2-back condition (T = 3.57; p = 011, small-volume FWE-corrected for amygdala ROI volume; see Figure 2B). Masking of this comparison with the F contrast for main effect of genotype revealed that there was a trend for a higher amygdala activation in the Pro homozygotes in all conditions, which was, however, negligible in the emotional 0-back condition and did not survive FWE-correction in any condition other than the emotional 2-back. A similar pattern was observed in extrastriate visuocortical structures, including the fusiform gyrus (BA 37), with Pro homozygotes showing a stronger activation of the fusiform face area in all four conditions (T = 3.28; p = 036, small-volume FWE corrected).

**Figure 3.Genotype-related:**
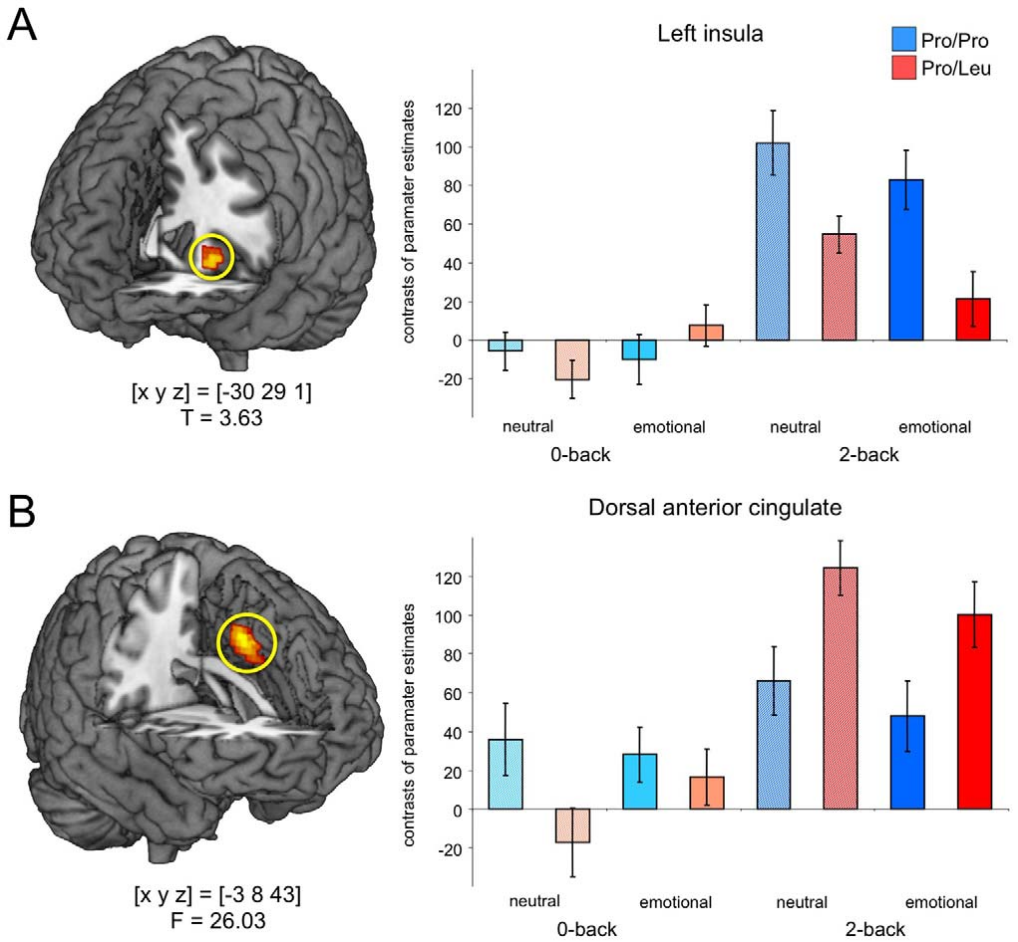
Genotype-related differences in insula and dACC activation. (**A**) There was a complex interaction of genotype condition (0-back vs. 2-back X neutral versus emotional faces) in the left anterior insula, with the most pronounced between-group difference observed for the emotional 2-back condition. (**B**) Leu carriers exhibited higher dACC activation in the 2-back condition when compared to Pro homozygotes, irrespective of emotional salience. Bar plots depict contrasts of parameter estimates and standard errors.

When the comparison of the genotype groups in the emotional 2-back condition was masked with the three-way interaction F contrast (genotype by N-back by emotional salience), the only brain region showing a reliable between-genotype difference was the left anterior insula (Figure 3B) with Pro homozygotes showing a stronger activation in both 2-back conditions, but the between-group difference more pronounced in the emotional 2-back condition (T = 3.63; p = 007, small-volume FWE corrected). A strong N-back by genotype interaction was observed in the dorsal ACC where Leu carriers showed a more pronounced activation in the 2-back condition, irrespective of emotional salience (F = 26.03; p = 045, whole-brain FWE corrected).

## Discussion

We had previously demonstrated that the AKAP5 Pro100Leu polymorphism modulates cognitive control of emotion processing, resulting in a more effective anger control and lower physical aggression in Leu carriers [39]. In a functional MRI experiment using a flanker task with task-irrelevant emotional faces in the background, Leu carriers showed stronger control of emotional interference as indexed by increased dACC activation, which in turn was associated with shorter reaction times during the flanker task. In the present study, we sought to extend those findings by assessing how cognitive processing of emotional stimuli is modulated when these stimuli are task-relevant themselves.

### AKAP5 Pro100Leu and working memory for emotional stimuli

Previous behavioral studies investigating effects of emotional face processing on working memory [42] have reported no consistent effect of emotional salience on performance accuracy in healthy subjects, but RTs in the 2-back task were slower for fearful than for neutral faces, a phenomenon that has been demonstrated to be particularly pronounced in individuals with high trait anxiety [40]. In our present study, we observed a marked genotype-dependent modulation of the amygdala response to face stimuli, which was markedly reduced in the Leu carriers in all conditions, but most prominently during the emotional 2-back condition. On the other hand, Leu carriers showed increased dACC activation in the 2-back condition. Evidence from twin or family studies suggests considerable heritability for both amygdala and anterior cingulate fMRI responses [60]–[62] and, given the careful matching of the groups and the compatibility of the results with those of our previous study [39], it seems conceivable that AKAP5 Pro100Leu modulates the cortical and amygdalar responses to emotional stimuli.

In contrast to our previous study, however, the genotype-related activation differences were associated with a performance advantage in Pro homozygotes rather than in the Leu carriers: While both experimental groups showed higher reaction times in the emotional relative to the neutral 2-back condition – an effect that has previously been linked to higher demands of attentional resources in the emotional 2-back task [40], [42], the RT increase was less pronounced in the Pro homozygotes. This pattern might at first seem paradoxical as increased amygdala reactivity has been linked to trait anxiety [63], and one might therefore expect longer rather than shorter RTs associated with higher amygdala reactivity. It should be noted, that the correlation of amygdala responses to emotional face stimuli with individual levels of (both state and trait) anxiety could be further modulated by attention, gaze direction and the task at hand [63]. AKAP5 Pro100Leu has thus far not been related to human anxiety, but has been shown to affect human aggression and anger, with Pro homozygotes showing higher physical aggression and lower anger control. Our results are thus compatible with the observation that a common polymorphism of the monoamine oxidase A (MAOA) gene that has been linked to human aggression has also been associated with an increased amygdala response to emotional faces [69].

The role of the amygdala in face processing has often been described in the context of a distributed face processing network that includes occipito-temporal structures specifically the fusiform face area (FFA). While both positive and negative emotions do indeed modulate the response of this entire network rather than just the amygdala [64], some authors argue that the increased response does not depend upon the emotional expression *per se*, but rather on the social relevance of emotional faces. Indeed, socially relevant features like gaze direction are also associated with amygdala activation [46], [65]. A further alternative view suggests that amygdala reactivity is, at a general level, modulated by facial distinctiveness, i.e. the deviation from a presumed “average face” [66].

Considering the amygdala as part of a distributed face processing network that responds to socially relevant or otherwise distinctive faces, one possible explanation for the observed increased amygdala activity and shorter RTs in Pro homozygotes might be an enhanced representation of the emotional faces that could facilitate task performance. Compatibly with this notion, Pro homozygotes showed increased activation not only in the amygdala, but also in the FFA. Efficient representation of emotional stimuli in a working memory task (indexed by initially high brain response followed by strong repetition suppression) has been linked to superior processing of emotional relative to neutral faces [67]. Notably, in that study, the processing advantage for emotional faces was not only observed in the amygdala, but also in the FFA. Given the increased activation of both of these structures in Pro homozygotes, we suggest that processing the stimulus (including its emotional salience) might beneficially affect performance in Pro homozygotes, whereas the weaker emotion-related amygdala response observed in Leu carriers might be detrimental. This notion is supported by the observation that in working memory tasks using faces as stimuli, FFA activity is a function of working memory load [68].

### Task-dependent modulation of emotion regulation by AKAP5 Pro100Leu

**Figure 4:**
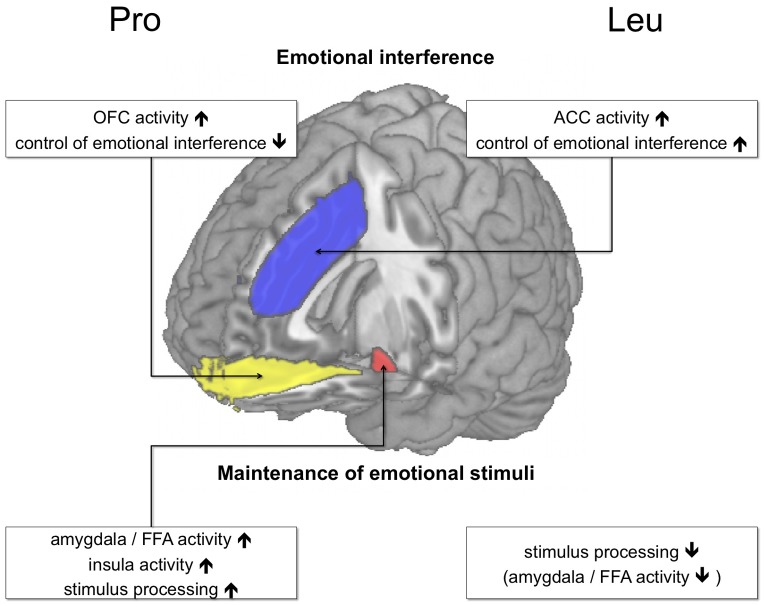
Effects of AKAP5 Pro100Leu on emotion processing (schematic overview). During emotional interference (see [39]), Pro homozygotes exhibit an increased OFC response (yellow) to task-irrelevant emotional distractors, possibly reflecting salience processing. Leu carriers, on the other hand, might suppress the emotional stimuli more efficiently as reflected by an increased ACC response (blue). When emotional stimuli are task-relevant (see results section), a different picture emerges, with Pro homozygotes exhibiting increased activity of the amygdala (and possibly a more extensive face processing network) and more efficient working memory performance.

The present study, along with our previous study showing more effective suppression of task-irrelevant emotional faces in Leu carriers, suggests that, at least in the case of AKAP5 Pro100Leu, a genetic modulation of emotion-cognition interactions might exert its effect in a task-dependent fashion. In our present experiment, the emotionally salient faces had to be actively kept in subjects’ working memory to perform the task successfully. Therefore, a weaker assessment of the emotional content would have presumably exerted deleterious effects on task performance. In this context, the increased dACC activation in the Leu carriers (Figure 3B) is noteworthy. Leu carriers had also shown such an increased dACC activation during emotional interference in an earlier study [39], but in that study, successful task performance required suppression of the emotional distracters, and was apparently beneficially influenced by the stronger dACC-dependent control of emotional processing in Leu carriers [39]. In the present study, on the other hand, Leu carriers might have allocated cognitive resources to dACC-dependent cognitive, control, and the dACC might have exerted a net inhibitory effect on the amygdala in Leu carriers, resulting in a suppression of emotion processing that was, given the task at hand, to some extent dysfunctional. Figure 4 summarizes the task-dependent effects of AKAP5 Pro100Leu on cognition-emotion interactions. The observed activation pattern in the insula, on the other hand, is more difficult to interpret. There was a generally increased activation of the left anterior insula in the 2-back condition (Table 2), but it was more pronounced in Pro homozygotes, with the between-group difference being most pronounced in the emotional 2-back condition (Figure 3A). We tentatively suggest that this complex pattern might be related to the role of the insula in the processing of both salience and aversive information [69], which might be more effective in the Pro homozygotes. Further research, however, is needed to specifically elucidate how AKAP5 Pro100Leu modulates the response of the human insula.

Imaging genetic studies of emotional face processing have thus far used several different paradigms, including face matching [70], emotion matching [71] or affect rating [72], but most have thus far not systematically distinguished between task-relevant and task-irrelevant face stimuli. There is, however, considerable behavioral and fMRI evidence that task-related processes exert profound influence on face perception [73]–[75]. Genetically mediated differences in face perception and emotion processing might also be task-dependent: While most studies investigating effects of the COMT Val108/158Met polymorphism on amygdala reactivity demonstrated higher amygdala activation in Val as compared to Met homozygotes [3], [17], [76], Domschke and colleagues recently observed the opposite pattern in a relatively large cohort [77] and suggested that stimulus material (aversive scenes versus faces), but also task differences might explain these apparently conflicting result. The task-dependence of amygdala reactivity has already been directly demonstrated for the 5-HTTLPR, which has repeatedly been associated with increased amygdala activation in carriers of the short allele [4], [18], [70], [77]. Schardt and colleagues [11] also replicated this pattern during passive viewing of emotional scenes, but showed that during voluntary emotion regulation no between-group differences could be observed. Together, these results, along with the results of our present study, suggest that future imaging genetics research should systematically address the task-specificity of genotype effects on behavior and brain activity patterns.

### Is AKAP5 Pro100Leu related to complex neurocognitive phenotypes?

AKAP5 is expressed throughout the human brain, including the amygdala, the hippocampus, and the striatum as well as distributed cortical regions like the anterior cingulate and the dorsolateral prefrontal and orbitofrontal cortices [38], [44], [78], [79]. While copy number variations of the AKAP5 gene have been implicated in the risk for schizophrenia and bipolar disorder [80], the Pro100Leu polymorphism has thus far not been associated with risk for major psychiatric disorders. In genetic studies of human psychiatric endophenotypes and personality traits, AKAP5 Pro100Leu has not been observed with genome-wide significance. In a recent metaanalysis of genotype-related individual differences in personality traits, however, the SNP was among the top hits for the Openness scale of the NEO-FFI [81], with Leu allele carriers showing higher scores in Openness. Because Openness and aggression are hardly correlated at all [82], it is difficult to directly relate that result to our findings that demonstrate an effect of AKAP5 Pro100Leu on aggression, anger, and emotion processing. It is, however, conceivable that the polymorphism might be related to apparently independent cognitive and affective processes, as has been observed for the well-studied COMT Val108/158Met polymorphism [83].

### Conclusions

Our results support previous evidence for a role of AKAP5 in PFC- and amygdala-dependent cognitive processing of emotional information [39]. They do, however, suggest that AKAP5 Pro100Leu does not uniformly modulate interactions between executive functions and emotional salience. Instead, genetically mediated differences in task performance appear to be modulated by the task-relevance of the emotional stimuli (Figure 3). Specifically, Leu carriers apparently exert higher cognitive control on, at least negative, emotions, which is reflected by a higher activation of the dACC [39], whereas Pro homozygotes might encode task-relevant emotional stimuli more effectively, resulting in better working memory performance for these stimuli. More generally our results point to the importance of task design in imaging genetics studies and suggest that apparently conflicting results in the research of genetically mediated individual differences in brain activity might at least in part be mediated by more or less subtle differences in the specific tasks employed.
